# Brain controllability and clinical relevance in schizophrenia

**DOI:** 10.1192/j.eurpsy.2022.516

**Published:** 2022-09-01

**Authors:** Q. Li, L. Luo, W. You, Y. Wang, Y. Wang, Q. Gong, F. Li

**Affiliations:** West China hospital of Sichuan university, Radiology, Chengdu, China

**Keywords:** schizophrénia, Resting-state functional magnetic resonance imaging, Recursive feature elimination, Controllability

## Abstract

**Introduction:**

Apart from the psychiatric symptoms, cognitive deficits are also the core symptoms of schizophrenia. Brain network control theory provided information on the role of a specific brain region in the cognitive control process, helping understand the neural mechanism of cognitive impairment in schizophrenia.

**Objectives:**

To characterize the control properties of functional brain network in first-episode untreated patients with schizophrenia and the relationships between controllability and psychiatric symptoms, as well as exploring the predictive value of controllability in differentiating patients from healthy controls (HCs).

**Methods:**

Average and modal controllability of brain networks were calculated and compared between 133 first-episode untreated patients with schizophrenia and 135 HCs. The associations between controllability and clinical symptoms were evaluated using sparse canonical correlation analysis. Support vector machine (SVM) and SVM-recursive feature elimination combined with the controllability were performed to establish the individual prediction model.

**Results:**

Compared to HCs, the patients with schizophrenia showed increased average controllability and decreased modal controllability in dorsal anterior cingulate cortex (dACC). Brain controllability predominantly in somatomotor, default mode, and visual networks was associated with the positive symptomatology of schizophrenia. The established model could identify patients with an accuracy of 0.68. Furthermore, the most discriminative features were located in dACC, medial prefrontal lobe, precuneus and superior temporal gyrus.

**Conclusions:**

Altered controllability in dACC may play a critical role in the neuropathological mechanisms of cognitive deficit in schizophrenia, which could drive the brain function to different states to cope with varied cognitive tasks. As symptom-related biomarkers, controllability could be also beneficial to individual prediction in schizophrenia.

**Disclosure:**

No significant relationships.

